# Incidence of Retinal Hemorrhage in Newborns With Intrauterine Growth Restriction (IUGR) at King Fahad Armed Forces Hospital, Jeddah

**DOI:** 10.7759/cureus.90960

**Published:** 2025-08-25

**Authors:** Faisal Alasmari, Husain A Alalgum, Amer M Alghamdi, Nourah A Alageel, Faris A Alhazmi, Adeeb Mogharbel, Manal Hadrawi

**Affiliations:** 1 College of Medicine, King Saud bin Abdulaziz University for Health Sciences, Jeddah, SAU; 2 Department of Ophthalmology, College of Medicine, King Saud University, King Saud University Medical City, Riyadh, SAU; 3 Department of Family Medicine, King Abdulaziz Medical City (KAMC) Jeddah, National Guard Health Affairs (NGHA), Jeddah, SAU; 4 Department of Ophthalmology, King Fahad Armed Forces Hospital, Jeddah, SAU; 5 Department of Medicine, King Abdulaziz University, Jeddah, SAU

**Keywords:** intrauterine growth restriction, iugr, neonate, retina, retinal hemorrhage

## Abstract

Objective

The objective of this study is to evaluate the incidence and associated risk factors of neonatal retinal hemorrhage (NRH) and, secondarily, to describe its clinical characteristics, laterality, and severity in newborns with intrauterine growth restriction (IUGR) from January 2017 to December 2021 at a tertiary center in Jeddah, Saudi Arabia.

Methods

This study was conducted using a retrospective chart review of all newborns with IUGR from January 2017 to December 2021 at King Fahad Armed Forces Hospital (KFAFH) in Jeddah, Saudi Arabia. Clinical records including demographics, neonatal as well as maternal factors, and ophthalmological exams were reviewed.

Results

Among 1775 newborns with IUGR, 62 (3.5%) newborns had positive findings of NRH. The incidence rate of NRH was 6.98 per 1000 live births (0.698%) per year. NRH was more commonly found bilaterally (36; 58.1%), in female newborns (43; 69.4%), and in those born through spontaneous vaginal delivery (SVD) (45; 72.6%). Most cases, 52 (83.9%), involved multiple retinal hemorrhage points rather than a single point (10; 16.1%). A higher gestational age (GA), which corresponds to a higher birth weight, was linked to an increased risk of retinal hemorrhages. The presence of jaundice, RDS, or neonatal hematological diseases was significantly associated with the development of NRH.

Conclusion

NRH is relatively common in infants with IUGR, usually resolving spontaneously without affecting vision. Its occurrence was associated with a higher GA, SVD, jaundice, respiratory distress syndrome, and hematological disorders. Targeted screening of IUGR infants with these risk factors may support early detection, though further research is needed to clarify prognostic and preventive strategies.

## Introduction

Neonatal retinal hemorrhage (NRH) is a common fundus finding in newborns within the first month of life [1], with reported incidence rates ranging from 2.6% to 50% [2,3]. Typically, NRH resolves on its own and does not impact the development of vision unless the macula is involved. If the macula is affected, it can lead to abnormal visual function and potentially disturb a child's quality of life. For example, delayed absorption of NRH can cause deprivation amblyopia due to an obscured visual axis. NRH can be categorized by its location (peripheral or posterior pole), affected layer (preretinal, intraretinal, or subretinal), and size [4].

The precise cause of NRH remains unclear, but some studies have identified associated risk factors. Nearly all studies agree that spontaneous vaginal delivery significantly increases the risk of NRH [5,6]. Other potential risk factors include a prolonged second stage of labor, advanced maternal age, and neonatal intracranial hemorrhage [5,7,8]. Although these studies investigated whether birth weight is a risk factor for NRH, the association was found to be insignificant. Only one study from Korea reported that three out of 27 newborns with intrauterine growth restriction exhibited NRH [9].

In our hospital, observational analysis revealed that a significant number of newborns with IUGR also had NRH. Therefore, this study aims to determine the incidence rate of NRH in neonates diagnosed with IUGR and secondarily to describe its clinical characteristics, laterality, and severity.

## Materials and methods

The Internal Review Board/Ethics Committee at King Fahad Armed Forces Hospital (KFAFH) approved this study. A retrospective chart review was conducted for all newborns with IUGR from January 2017 to December 2021 at King Fahad Armed Forces Hospital in Jeddah, Saudi Arabia. The total number of IUGR newborns was used to calculate the incidence of retinal hemorrhage in these newborns. A convenient sampling technique with a 2:1 ratio (Controls: Cases) was used to select the control group (IUGR newborns without NRH) for comparison with the case group (IUGR newborns with NRH).

All newborns who had IUGR and were also small for gestational age (SGA) at King Fahad Armed Forces Hospital, Jeddah, Saudi Arabia from January 2017 to December 2021 were included in the study. However, newborns with birth trauma, incomplete data, and extremely preterm infants (<31 weeks) were excluded. The charts were reviewed to collect data on gender, gestational age (GA), type of delivery, birth weight, birth weight percentile, Apgar scores, neonatal/maternal factors, and the incidence and characteristics of NRH (laterality, location, type, number, retinal level, size, severity, and macula or optic disc involvement). All IUGR cases underwent a retinal examination by an ophthalmologist within a maximum of 120 hours (five days) of birth and before discharge from the neonatal intensive care unit, using an indirect ophthalmoscope after pupil dilation.

Data collected was entered into Microsoft Excel and then exported to IBM SPSS Statistics for Windows, Version 22 (Released 2013; IBM Corp., Armonk, New York, United States) for statistical analysis. Quantitative data were presented as mean values and standard deviations, while qualitative data were presented as frequencies and percentages. The chi-square test was used to compare categorical variables, with p-values of < 0.05 considered statistically significant.

## Results

Among a total of 1775 newborns with IUGR found from January 2017 to December 2021, 62 (3.5%) newborns manifested findings of NRH. The incidence of NRH was calculated to be 6.98 per 1000 live births with IUGR (0.698%) per year (Table 1).

**Table 1:** Incidence of the NRH in IUGR NRH: Neonatal Retinal Hemorrhage; IUGR: Intrauterine Growth Restriction

Variable	In one year	In five years	Per 1000 live births with IUGR/1 year
Incidence	0.006986	0.03493	6.985915

A representative population of all newborns with IUGR was selected by the convenience sampling technique using a ratio of 2:1 (124 Controls: 62 Cases) to compare cases with NRH and those without NRH. A total of 186 newborns with IUGR have been reviewed, with 84 (45.2%) being male and 102 (54.8%) being female. Nearly half of the newborns (90, 48.4%) were delivered through spontaneous vaginal delivery (SVD), while the other half (96, 51.6%) were delivered via cesarean section (CS). Of those born through SVD, no one had trauma related to childbirth, such as edema of the child's head, subcutaneous hemorrhage, or intrathecal hematoma, and no injuries were documented after birth.

The mean GA at birth was 36.65 weeks (SD = 3.09), whereas the mean birth weight was 1.96 kg (SD = 0.52). At the same time, the mean percentile score was 5.30 (SD = 1.30), and the mean Apgar scores at one minute and five minutes after birth were 7.25 (SD = 1.21) and 8.68 (SD = 0.67), respectively. Moreover, 62 (3.5%) newborns with IUGR were found to have NRH in the dilated fundus exam, and 45 (72.6%) of them were born by SVD (Table 2).

**Table 2:** Analysis of Hemorrhage in Newborns: Gender, Delivery Type, and Associated Variables HG: Hemorrhage; GA: Gestational Age; BW: Birth Weight; SD: Standard Deviation

Variable		Total	Absence of HG	Presence of HG	p-value
Gender (n,%)	Male	84 (45.2%)	65 (52.4%)	19 (30.6%)	0.005
	Female	102 (54.8%)	59 (47.6%)	43 (69.4%)	
Type of Delivery (n,%)	Spontaneous vaginal delivery	90 (48.4%)	45 (36.3%)	45 (72.6%)	<0.001
	Cesarean section	96 (51.6%)	79 (63.7%)	17 (27.4%)	
GA (Mean, SD)		36.65 (3.09)	36.5 (3.30)	36.95 (2.63)	0.349
BW (Mean, SD)		1.96 (0.52)	2 (0.57)	1.89 (0.40)	0.194
Percentile (Mean, SD)		5.30 (1.30)	5.44 (1.36)	5.01 (1.13)	0.032
Apgar at 1 min (Mean, SD)		7.25 (1.21)	7.23 (1.20)	7.29 (1.22)	0.766
Apgar at 5 min (Mean, SD)		8.68 (0.67)	8.65 (0.69)	8.76 (0.64)	0.273

**Figure 1:**
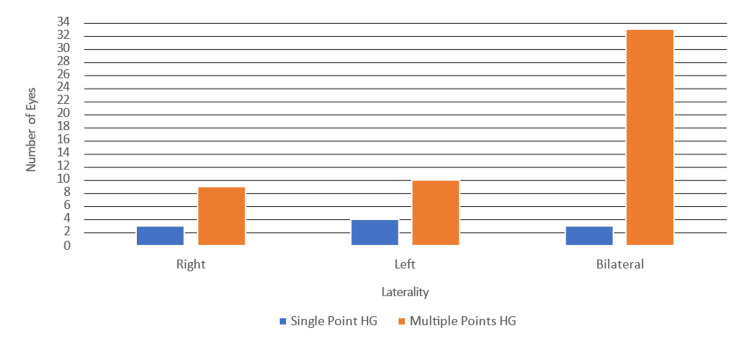
Frequency of Retinal Hemorrhages by Eye and Severity (Single Point vs. Multiple Points) HG: Hemorrhage

In terms of the laterality (affected eye) of retinal hemorrhages, NRH was more frequent bilaterally compared to affecting only one eye as 12 (19.4%) newborns had NRH in the right eye, 14 (22.6%) newborns had NRH in the left eye, and 36 (58%) newborns had NRH in both eyes. Additionally, the vast majority (52, 83.9%) were found to have multiple retinal hemorrhage points rather than only one single hemorrhage point (10, 16.1%) as shown in Table 3 and Figure 1. All examined newborns exhibited no signs of uveitis, retinitis, or optic nerve pathologies.

**Table 3:** Laterality and Severity of Retinal Hemorrhages

Variable		n	%
Affected Eye	Right	12	19.4%
	Left	14	22.6%
	Bilateral	36	58.1%
Hemorrhage Point	Single	10	16.1%
	Multiple	52	83.9%

Additionally, this study examined factors associated with the occurrence of retinal hemorrhages in newborns with IUGR using logistic regression (Table 4). It was found that a higher GA was linked to an increased risk of retinal hemorrhages, with each additional week of GA raising the odds by 51% (Exp(B) = 1.51, 95% CI: 1.13 - 2.02). Conversely, lower birth weight (BW) was significantly associated with a reduced risk of retinal hemorrhages, where a decrease of one kilogram in BW corresponded to a 97% reduction in the odds (Exp(B) = 0.03, 95% CI: 0.00 - 0.21). Furthermore, CS delivery, compared to SVD, was associated with a substantially lower risk of retinal hemorrhages, reducing the odds by 87% (Exp(B) = 0.13, 95% CI: 0.06 - 0.32). In contrast, BW percentile score, gender, and Apgar scores at 1 and 5 minutes did not show statistically significant associations with the occurrence of retinal hemorrhages in this analysis.

**Table 4:** Factors Determining the Occurrence of Retinal Hemorrhage B: Regression Coefficient; Wald: Wald Statistic; Sig.: Significance (p-value); Exp(B): Exponentiated B; C.I.: Confidence Interval

Variable	B	Wald	Sig.	Exp(B)	95% C.I.for EXP(B)	
					Lower	Upper
GA	0.41	7.86	0.01	1.51	1.13	2.02
BW	-3.58	11.90	<0.01	0.03	0.00	0.21
Percentile	0.09	0.22	0.64	1.10	0.74	1.63
Gender (1)	0.55	2.12	0.15	1.73	0.83	3.64
Apgar at 1 min	-0.23	1.05	0.31	0.79	0.51	1.24
Apgar at 5 min	0.38	0.73	0.39	1.47	0.61	3.52
Type of Delivery (1)	-2.01	20.36	<0.01	0.13	0.06	0.32
Constant	-10.48	3.84	0.05	0.00	-	-

The association between various neonatal and other associated conditions and the presence of retinal hemorrhage was analyzed using the chi-square test. Firstly, newborns with jaundice were significantly more likely to have NRH compared to those without jaundice (p = 0.005). Secondly, the presence of respiratory distress syndrome (RDS) or other respiratory diseases was significantly associated with a higher risk of NRH (p < 0.001). Thirdly, neonates with diagnosed hematological diseases had a significantly higher prevalence of NRH compared to those without these diseases (p = 0.021). Fourthly, having a specific diagnosed syndrome was significantly associated with an increased risk of retinal hemorrhage (p = 0.012). Lastly, mothers with other comorbid medical conditions were significantly more likely to have neonates with NRH (p = 0.013). However, maternal diabetes or neonatal cardiac, central nervous system, hormonal, metabolic, renal, hepatobiliary, gastrointestinal, septic diseases, or twin-to-twin transfusion syndrome did not show statistically significant associations with NRH in our analysis (Table 5).

**Table 5:** Association between Neonatal and Other Associated Conditions and Retinal Hemorrhage CNS: Central Nervous System; RDS: Respiratory Distress Syndrome; GI: Gastrointestinal

Associated Diseases		Absence of HG		Presence of HG		p-value
		n	%	n	%	
Jaundice	No	82	66.1%	53	85.5%	0.005
	Yes	42	33.9%	9	14.5%	
Congenital Cardiac	No	117	94.4%	57	91.9%	0.527
	Yes	7	5.6%	5	8.1%	
CNS Related	No	117	94.4%	55	88.7%	0.169
	Yes	7	5.6%	7	11.3%	
Hormonal and Metabolic	No	116	93.5%	62	100.0%	0.054
	Yes	8	6.5%	0	0.0%	
Renal, Electrolytes, pH Imbalance	No	111	89.5%	60	96.8%	0.087
	Yes	13	10.5%	2	3.2%	
RDS and Other Respiratory	No	81	65.3%	57	91.9%	<0.001
	Yes	43	34.7%	5	8.1%	
Hepatobiliary and GI	No	116	93.5%	61	98.4%	0.147
	Yes	8	6.5%	1	1.6%	
Hematological	No	102	82.3%	59	95.2%	0.021
	Yes	22	17.7%	3	4.8%	
Sepsis	No	114	91.9%	60	96.8%	0.343
	Yes	10	8.1%	2	3.2%	
Others	No	112	90.3%	53	85.5%	0.335
	Yes	12	9.7%	9	14.5%	
Specific Syndromes	No	124	100.0%	58	93.5%	0.012
	Yes	0	0.0%	4	6.5%	
Maternal Diabetes	No	119	96.0%	61	98.4%	0.665
	Yes	5	4.0%	1	1.6%	
Other Maternal Conditions	No	108	87.1%	61	98.4%	0.013
	Yes	16	12.9%	1	1.6%	
Twin-to-Twin Transfusion Syndrome	No	122	98.4%	62	100.0%	0.553
	Yes	2	1.6%	0	0.0%	

Next, logistic regression analysis identified several variables significantly associated with NRH in IUGR cases. Firstly, the odds of NRH were nearly four times higher in newborns with jaundice compared to those without jaundice (Exp(B) = 4.01, 95% CI: 1.52 - 10.59). Secondly, newborns with renal, electrolyte, or pH imbalances had a tenfold higher likelihood of developing NRH compared to those without these imbalances (Exp(B) = 10.30, 95% CI: 2.00 - 52.98). Thirdly, the presence of respiratory distress syndrome (RDS) or other respiratory issues increased the odds of NRH by eight times (Exp(B) = 8.39, 95% CI: 2.79 - 25.19). Fourthly, newborns diagnosed with hematological diseases were over five times more likely to have NRH than those without such diseases (Exp(B) = 5.58, 95% CI: 1.42 - 21.97). Lastly, mothers with medical conditions other than diabetes had a 26-fold higher chance of having babies with NRH compared to mothers without these conditions (Exp(B) = 26.47, 95% CI: 3.16 - 221.79) (Table 6).

**Table 6:** Neonatal and Other Associated Conditions That May Predict Retinal Hemorrhage CNS: Central Nervous System; RDS: Respiratory Distress Syndrome

Associated Conditions	B	Wald	Sig.	Exp(B)	95% C.I. for EXP(B)	
					Lower	Upper
Jaundice	1.39	7.86	0.01	4.01	1.52	10.59
Congenital Cardiac	0.90	1.37	0.24	2.47	0.54	11.16
CNS	-0.14	0.04	0.84	0.87	0.21	3.55
Hormonal and Metabolic Imbalance	21.22	0.00	1.00	> 100	0.00	0.00
Renal, Electrolytes, and pH Imbalance	2.33	7.78	0.01	10.30	2.00	52.98
RDS and Other Respiratory	2.13	14.37	0.00	8.39	2.79	25.19
Hepatobiliary and GI	2.38	4.42	0.04	10.85	1.18	100.06
Hematological	1.72	6.04	0.01	5.58	1.42	21.97
Infection or Sepsis	1.61	1.87	0.17	4.99	0.50	50.00
Others	0.12	0.04	0.84	1.13	0.35	3.65
Specific syndromes	-21.59	0.00	1.00	0.00	0.00	0.00
Maternal diabetes	1.31	1.02	0.31	3.71	0.29	47.22
Other maternal conditions	3.28	9.12	0.00	26.47	3.16	221.79
Twin-to-twin Transfusion Syndrome	19.09	0.00	1.00	> 100	0.00	0.00

## Discussion

NRH is a common neonatal fundus manifestation occurring in newborns within one month of birth [1]. The reported incidence varies from 2.6% to 50% [2,3]. In a recent ophthalmological screening of healthy newborns using a wide-angle digital imaging device, 27.89% were found to have retinal hemorrhage [10]. Notably, only one Korean study has addressed the incidence of NRH in IUGR newborns, reporting three cases of NRH among 27 newborns with intrauterine growth restriction [9]. In our study, among 1775 newborns with IUGR, 62 (3.5%) showed positive findings of NRH, resulting in an incidence rate of 6.98 per 1000 live births per year in IUGR newborns.

Similar to the retinal findings observed in healthy newborns [4], NRH in IUGR cases was typically bilateral, predominantly intraretinal, and characterized by multiple focal points located in the posterior pole. It is known that the prevalence of NRH is lower among neonates delivered via CS and higher among those with cranial hematoma [7]. Our study confirmed a significant correlation between CS delivery and a reduced risk of NRH. Additionally, none of the newborns delivered through SVD had documented childbirth-related trauma or post-birth injuries.

The average GA of IUGR newborns with NRH in our study was 36.95 weeks, slightly lower than the 37+6 weeks reported in the Korean study. However, in both studies, IUGR newborns without NRH had an average GA of 36.5 weeks, supporting our finding that higher GA is associated with an increased risk of NRH. Specifically, each additional week of GA increased the odds of NRH by 51%.

The average BW in IUGR newborns with NRH was 1.89 kg in our study, compared to 2 kg in the Korean study. We found that lower BW was significantly associated with a reduced risk of NRH, with a decrease of one kilogram in BW reducing the odds of NRH by 97%. Additionally, our study found that newborns with jaundice were significantly more likely to have NRH. Other significant factors associated with NRH included the presence of RDS and neonatal hematological diseases, which were not addressed in other studies.

Moreover, NRH in IUGR cases predominantly presented in the posterior pole, with 83.9% of cases involving multiple focal points of hemorrhages rather than a single point.

Limitations

Our study has some limitations. First, its retrospective design relied on existing medical records, which were sometimes incomplete or handwritten, limiting the availability of detailed data on retinal hemorrhage characteristics and involvement of the macula or optic disc. Second, being a single-center study, the findings may not be generalizable to other populations or healthcare settings. Third, several odds ratios (e.g., renal/pH imbalance, maternal comorbidities) were accompanied by wide confidence intervals, reflecting small subgroup sizes and resulting in unstable estimates; these should therefore be considered exploratory. Fourth, because NRH type (intraretinal, preretinal, subretinal) and macular involvement were not systematically documented, and follow-up examinations were inconsistently available, our conclusions are based on baseline screening findings and do not extend to long-term prognosis. Finally, the lack of long-term follow-up restricted our ability to assess the natural course and visual outcomes of NRHs. Future multicenter prospective studies with standardized protocols are warranted to validate and expand upon these findings.

## Conclusions

NRH is a relatively common finding among newborns with IUGR. In most cases, NRH resolves spontaneously and does not interfere with visual development; however, its occurrence highlights the need for careful ophthalmological evaluation. Our study demonstrated significant associations between NRH and factors such as higher GA, SVD, jaundice, RDS, and neonatal hematological disorders. These findings suggest that targeted screening of IUGR infants, particularly those with additional risk factors, may aid in early detection and management. Future prospective studies are warranted to further characterize the severity, prognostic implications, and potential preventive strategies for NRH in IUGR newborns.
